# Methylome analysis identifies a Wilms tumor epigenetic biomarker detectable in blood

**DOI:** 10.1186/s13059-014-0434-y

**Published:** 2014-08-19

**Authors:** Jocelyn Charlton, Richard D Williams, Mark Weeks, Neil J Sebire, Sergey Popov, Gordan Vujanic, William Mifsud, Marisa Alcaide-German, Lee M Butcher, Stephan Beck, Kathy Pritchard-Jones

**Affiliations:** UCL Institute of Child Health, University College London, 30 Guilford Street, London, WC1N 1EH UK; The Institute of Cancer Research, 15 Cotswold Road, Sutton, Surrey SM2 5NG UK; Department of Pathology, Cardiff University School of Medicine, Heath Park, Cardiff, CF14 4XN UK; UCL Cancer Institute, University College London, 72 Huntley Street, London, WC1E 6BT UK

## Abstract

**Background:**

Wilms tumor is the most common pediatric renal malignancy and there is a clinical need for a molecular biomarker to assess treatment response and predict relapse. The known mutated genes in this tumor type show low mutation frequencies, whereas aberrant methylation at 11p15 is by far the most common aberration. We therefore analyzed the epigenome, rather than the genome, to identify ubiquitous tumor-specific biomarkers.

**Results:**

Methylome analysis of matched normal kidney and Wilms tumor identifies 309 preliminary methylation variable positions which we translate into three differentially methylated regions (DMRs) for use as tumor-specific biomarkers. Using two novel algorithms we show that these three DMRs are not confounded by cell type composition. We further show that these DMRs are not methylated in embryonic blastema but are intermediately methylated in Wilms tumor precursor lesions. We validate the biomarker DMRs using two independent sample sets of normal kidney and Wilms tumor and seven Wilms tumor histological subtypes, achieving 100% and 98% correct classification, respectively. As proof-of-principle for clinical utility, we successfully use biomarker DMR-2 in a pilot analysis of cell-free circulating DNA to monitor tumor response during treatment in ten patients.

**Conclusions:**

These findings define the most common methylated regions in Wilms tumor known to date which are not associated with their embryonic origin or precursor stage. We show that this tumor-specific methylated DNA is released into the blood circulation where it can be detected non-invasively showing potential for clinical utility.

**Electronic supplementary material:**

The online version of this article (doi:10.1186/s13059-014-0434-y) contains supplementary material, which is available to authorized users.

## Background

Wilms tumor (WT) is the most common pediatric renal cancer with a prevalence of one in 10,000 children [1]. In Europe, most patients receive four weeks of pre-operative chemotherapy prior to complete or partial nephrectomy, followed by tumor stage and histology-dependent post-operative treatment [2]. Although overall survival rates are good, there is a clinical need for a biomarker to evaluate patient response to chemotherapy and improve prediction of relapse.

Circulating cell-free DNA (cfDNA) isolated from blood has been used to assess tumor burden in other cancers [3–6]. In WT, the few genes that are recurrently mutated show low mutation frequencies - *WTX* (18%) [7], *CTNNB1* (15%) [8] and *WT1* (12%) [8] - and do not account for the majority of WTs. However, epimutation affecting the *IGF2*/*H19* locus at 11p15.5 is much more common (69%) [8]. Additional genes and regions known to be affected by methylation in WT include *GLIPR1* [9], imprinted genes *NNAT* [10] and the *WT1*-antisense region [11], various satellite regions [12,13], *HACE1* [14], *RASSF1A* [15], *P16* and the protocadherin cluster at 5q31 [16]. Consequently, we concluded that interrogation of the methylome rather than the genome may be more likely to reveal ubiquitous tumor-specific biomarkers. Therefore, we performed genome-wide methylome analysis of matched WT and surrounding normal kidney (NK) to identify WT-specific sites of methylation which we then assessed in cfDNA for use as WT biomarkers.

## Results

**Figure 1:**
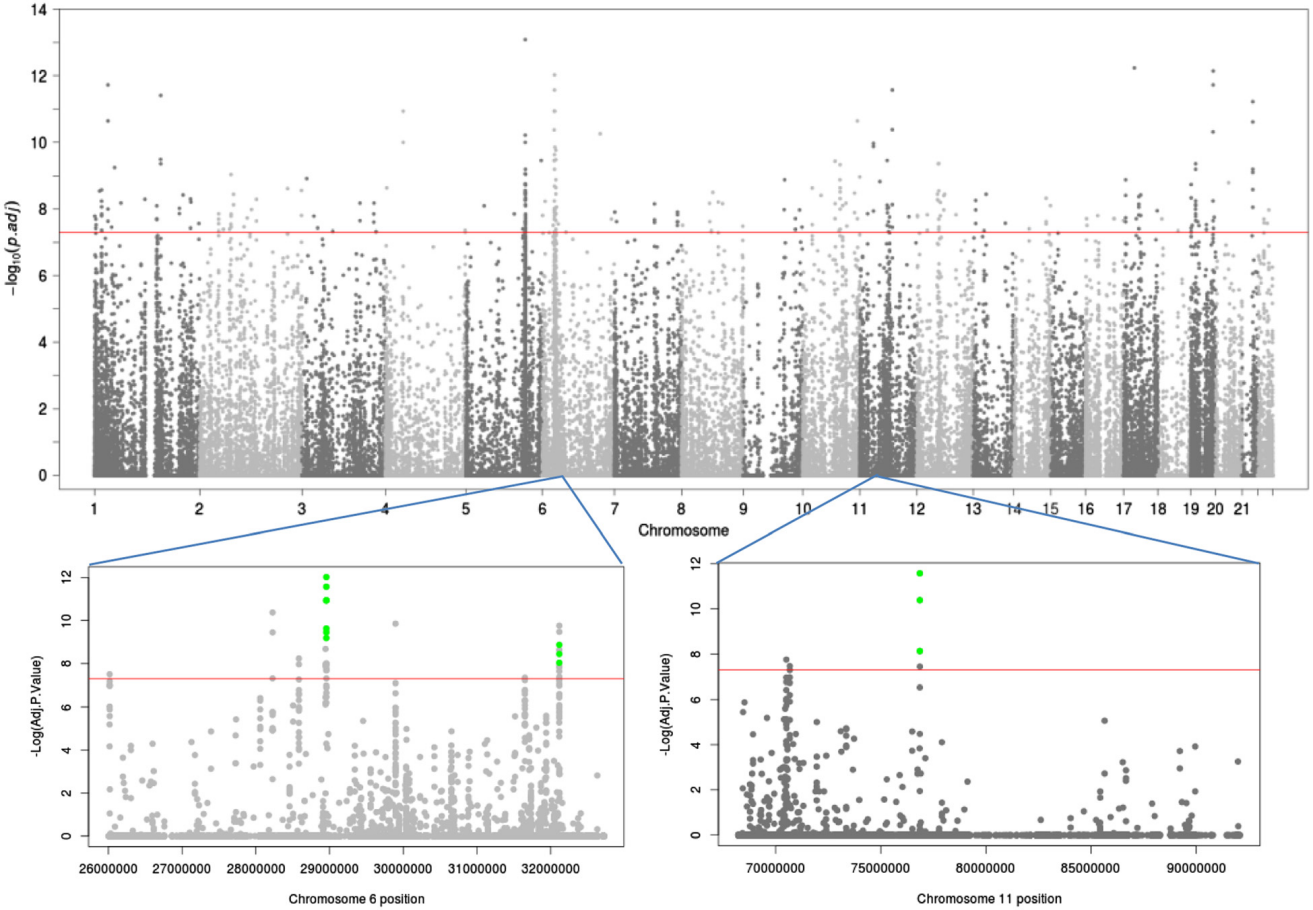
**Identification of differentially methylated loci with genome-wide significance.** Manhattan plot showing -log_10_ *P*-values for all CpGs (462,537) generated by linear modeling of normal kidney (NK) against Wilms tumor (WT). CpGs with genome-wide significance (n = 309) appear above the red line. DMR-1 and DMR-2 on chromosome 6 and DMR-3 on chromosome 11 are shown in green.

To identify tumor-specific methylation variable positions (MVPs), as previously defined [17], we derived methylation levels (β; 0 = unmethylated to 1 = methylated) for 462,537 CpG sites at single base-pair resolution using the Illumina Infinium HumanMethylation450 platform and performed linear modeling to compare 22 matched pairs of NK and WT (full clinical details in Additional file 1). We identified 309 MVPs of genome-wide significance (*P* < 5 × 10^-8^; Figure 1). Due to the matched study design there was no need to adjust for age, race or gender and we can exclude the possibility of MVPs being confounded by genetic polymorphism(s). We then applied the novel pipeline Lasso, recently developed for analysis of Illumina 450 k data, which considers the local CpG density to group MVPs into functionally more relevant differentially methylated regions (DMRs) [18]. Using this method, we identified three DMRs which were hypermethylated in WT with respect to NK (Table 1).

**Table 1:** **Differentially methylated regions hypermethylated in Wilms tumor compared to normal kidney**

**DMR**	**Location**	**First CpG**	**Last CpG**	**Size (bp)**	**Number of CpGs**	**DMR** ***P*** **-value**	**CpG island**	**Nearest gene**
1	6p22.1	28956226	28956426	200	8	1.58E-10	CpG:42	ZNF311
2	6p21.32	32116905	32116963	58	3	4.67E-09	CpG:56	*PRRT1*
3	11q13.5	76858947	76859056	109	3	2.48E-09	CpG:38	*MYO7A*

To assess possible confounding due to differential cell type composition, we carried out two analyses. First, we conducted histological analysis confirming our samples to be composed of the expected major cell types, consisting of 95% epithelia and 5% stroma in NK while WT showed varying proportions of immature stroma, epithelia and blastema (Figure S1 in Additional file 2). Second, we used a recently published algorithm, RefFreeEWAS [19], which corrects *P*-values based on estimated cell type contributions. Performing linear modeling using this algorithm, we identified 7,272 CpGs with genome-wide significance (*P* < 5 × 10^-8^) of which 937 had Δβ > 0.3 and were therefore considered cell composition-corrected MVPs. Of these, 766 were hyperMVPs and 171 were hypoMVPs in WT with respect to NK (Additional file 3). For hyperMVPs in particular, we saw a striking positive enrichment for location within CpG islands (+18% compared to background). There were 483 CpG islands targeted by aberrant methylation in total (Additional file 4) with a varying number of corrected MVPs per island. The greatest enrichment of MVPs occurred in two CpG islands on chromosome 6 (CpG:56 and CpG:42) with 13 and 11 MVPs, respectively, which overlapped with DMR-1 and -2. Using a threshold of ≥3 MVPs per DMR, we then mapped the cell type-corrected MVPs onto the DMRs identified with Lasso, confirming that all three DMRs were not confounded by cell composition effects. We therefore continued our analysis focusing on DMR-1 to -3.

**Figure 2:**
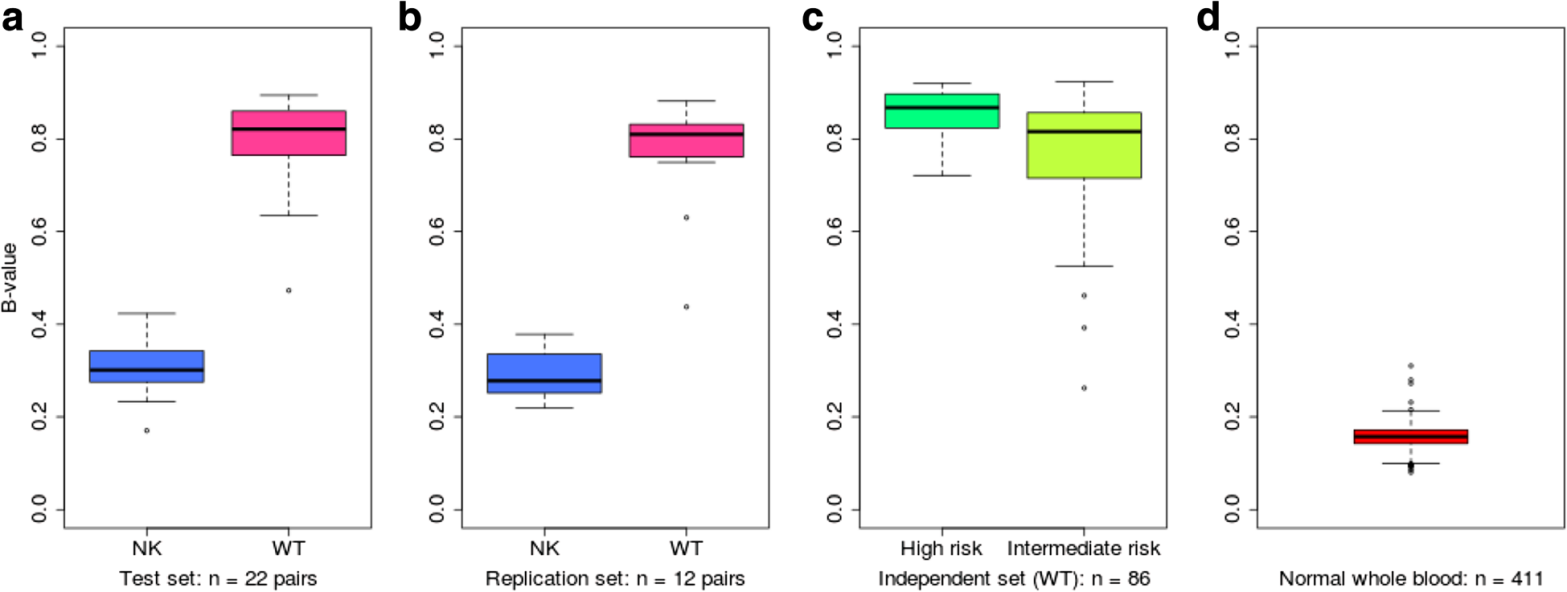
**Methylation levels for DMR-1 to -3 significantly distinguish normal kidney from Wilms tumour and whole blood. (a,b)** Aggregated methylation levels across DMR-1 to -3 significantly separate normal kidney (blue) and Wilms tumor (pink) in the test set (n = 22 pairs, *P* = 3.85 × 10^-17^) **(a)** and in the replication set (n = 12 pairs, *P* = 1.47 × 10^-9^) **(b)**. **(c)** Methylation levels were high in an independent dataset of WT including high risk (dark green, n = 25) and intermediate risk histological subtypes (light green, n = 61). High risk WT showed significantly higher methylation levels than intermediate risk WT (*P* = 0.0024). **(d)** Whole blood (n = 411) shows low methylation levels (red).

Aggregating DNA methylation across DMR-1 to -3 (β_mean_), we found that WT had significantly greater levels of methylation with respect to NK for the discovery dataset as well as an independent dataset of 12 pairs (*P* = 3.85 × 10^-17^ and *P* = 9.26 × 10^-10^, respectively, two-tailed *t*-test; Figure 2a,b). Furthermore, β_mean_ was consistently high across an independent cohort of fresh frozen WT (n = 86; Table S4 in Additional file 2) encompassing the seven post-chemotherapy WT histological subtypes classified into two risk groups, as defined by the International Society of Paediatric Oncology [20] (SIOP; Figure 2c). Within this cohort, a significant difference was seen between WT risk groups (*P* = 0.0024, 2-tailed T-test) with more elevated methylation levels observed in high risk WT (average β_mean_ = 0.87 vs. 0.78). Based on methylation levels in the discovery dataset, a support vector machine (using R package e1071) correctly classified 100% and 98% samples within each replication set respectively indicating the discriminative potential of these DMRs as biomarkers. Clinical details for both of these cohorts can be found in Additional file 1: Table S1.

DMR-1 and -2 are located within the extended major histocompatibility complex (MHC) region [21]. Although the MHC is highly polymorphic [22], our matched study design controlled for any genetic heterogeneity, ensuring that the observed signal was not confounded by copy number or other DNA sequence variation. We validated methylation levels for these DMRs using bisulfite-sequencing (Figure S3 and Table S5 in Additional file 2) and confirmed the absence of C > T mutation. MHC cluster hypermethylation and reciprocal loss of gene expression is common across cancers as a mechanism to evade immunosurveillance and increase oncogenic potential [23–26]. To further explore the association between DMR methylation and tumorigenesis, we extracted DNA from three specimens of human embryonic kidney (EK; gestational age = 22, 22 and 23 weeks) and separately microdissected embryonic blastema (EB; n = 3; the predicted WT cell of origin). Bisulfite sequencing of DMR-1 and -2 showed average β-values of 0.007 for EK and 0.12 for EB. Furthermore, analysis of methylation levels in 20 cases with matched WT precursor lesions termed nephrogenic rests (NRs) showed intermediate methylation levels (Figure S2 in Additional file 2). Put together, these data suggest that sequential increase in methylation levels is associated with transformation of embryonic precursor cells towards a malignant phenotype.

As all DMRs were methylated in WT in comparison to NK, we predicted that levels of methylated DNA in the circulation may increase with tumor burden. When assessing levels of a methylated blood biomarker, varying proportions of leukocyte subpopulations can alter the overall methylation signal, giving false yield [27]. Therefore, to assess the potential influence that blood populations may have on our dataset, we examined publically available methylation signatures that define normal peripheral blood subgroups [28] as well as methylation levels of normal whole blood (extracted using Marmal-aid [29]; n = 411). We found that DMR-1 to -3 did not overlap with any blood-related methylation signature, and that normal blood methylation levels for DMR-1 to -3 were extremely low (average β_m_ = 0.12; Figure 2d). Therefore, we concluded that the WT-specific hypermethylated DMRs were not detected as a result of shifts in leukocyte populations in chemotherapy-treated tissue. Hence, we explored the potential of DMR-1 to -3 as tumor-specific blood biomarkers, as they should be detectable above a low background and should not be confounded by shifts in leukocyte populations in the circulation.

**Figure 3:**
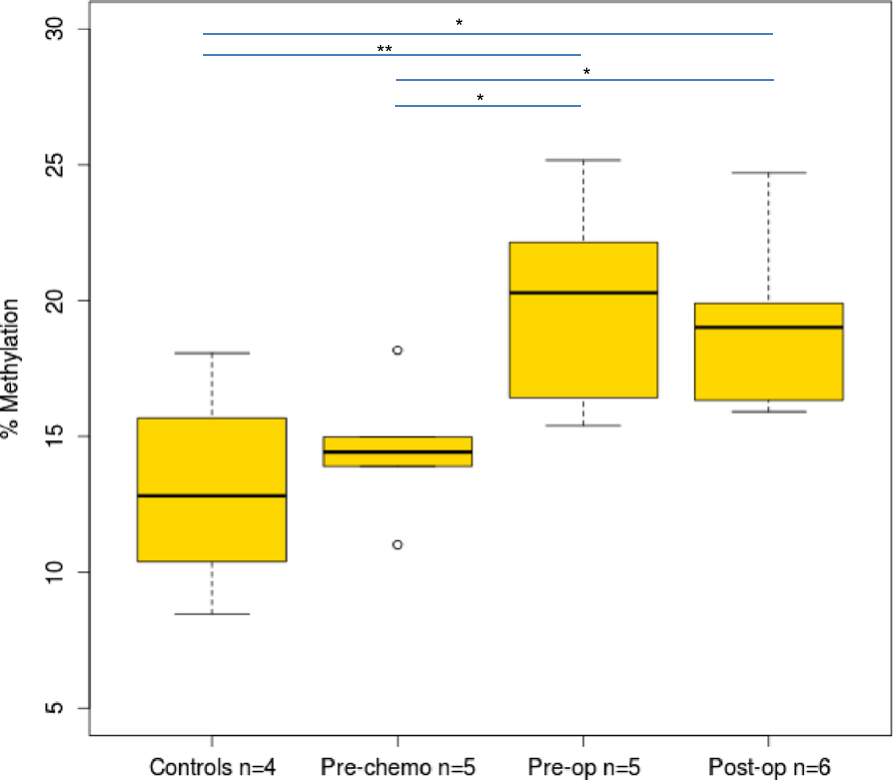
**Levels of methylation in serum cfDNA during WT treatment.** Levels of %M_mean_ (for 14 CpGs within DMR-2) show significant differences between controls and pre-op samples (*P* = 0.010), controls and post-op samples (*P* = 0.017), pre-chemotherapy and pre-op (*P* = 0.028) and pre-chemotherapy and post-op (*P* = 0.047; all by two-tailed *t*-test). Data show increasing levels of methylated cfDNA associated with WT necrosis. Level of significance is shown asterisks (*P < 0.05; **P < 0.01) with horizontal lines indicating the respective comparison.

To test the potential efficacy of these biomarkers, we performed bisulfite-sequencing of DMR-2 on cfDNA isolated from serum samples taken from 10 children with WT at diagnosis, during pre-operative chemotherapy and following nephrectomy as well as four cancer-free age-matched control serum samples (Table S6 in Additional file 2). The entire sequenced region (chr6:32,116,940-32,117,259) spanned 319 bp and included 44 CpGs, many of which showed either no variation or extreme variation in methylation between samples. Therefore, to identify CpGs that showed consistent methylation at one time-point and differential methylation between time-points, we grouped samples within each time-point and performed an ANOVA test. From this, we selected a subset of CpGs (n = 14) that showed differential methylation between at least one pair of groups, irrespective of directionality of methylation changes, avoiding those with very low variance. Aggregating the percentage of methylated cfDNA across these 14 CpGs (%M_mean_) showed that control samples had the lowest levels of methylated cfDNA (13.4) and that children with WT had relatively higher levels taken at diagnosis before treatment starts (14.5). There was then a substantial and significant increase in the level of methylated cfDNA taken after the pre-operative chemotherapy phase (19.9) that persisted into the immediate post-operative period (19.2) (Figure 3). This proof-of-principle experiment establishes DMR-2 as a potential blood-based biomarker for WT.

## Discussion

This study has identified three DMRs with genome-wide significance in WT that are present in nearly all WT (118 out of a total of 120 WTs examined) and are not mediated by cell type composition. In comparison to previous mutational studies, our epigenetic biomarker is far more ubiquitous and even supersedes 11p15 epimutation (found in approximately 70% of WT cases) as the most common biomarker in WT. Therefore, our results suggest that analysis of an epigenetic mark in cfDNA could be more successful than analysis of tumor-specific somatic mutations, which are much less common. Furthermore, our study revealed a significant difference in methylation levels between intermediate and high risk WT which could be of diagnostic use and suggest a use for novel therapeutic approaches to high risk WT.

Although the NK samples do not contain blastema but WT samples do, our correction for cell type composition compensates for these effects, allowing for identification of three true DMRs that are not mediated by cell mixture effects. Methylation analysis of DMR-1 and -2 in EK and EB showed that these regions are not methylated in the presumed WT cell of origin. Analysis of NRs showed that the precursor lesion had intermediate levels of methylation at the same regions which were then further methylated in the associated tumors (Figure S2 in Additional file 2). We therefore concluded that increased methylation at these loci is associated with tumorigenic transformation and is not simply a manifestation of the embryonic origin of these tumors. Furthermore, as 84/86 WTs were correctly classified by methylation status in our independent cohort, which included tumors stratified into all seven histological subtypes, and consistent high levels of methylation were seen in the test set of tumors with detailed assessment of their variable cellular composition (Figure S1 in Additional file 2), we can conclude that all cells constituting the tumor contributed to the observed gain of methylation.

Our data suggest that WT release methylated DMR-2 cfDNA (M-cfDNA) following exposure to chemotherapy. Supporting this, a higher proportion of M-cfDNA (%M_mean_ of 23.6 compared to 17.3) was observed in serum sampled after pre-operative chemotherapy in patients with the regressive subtype of WT, defined by more than two-thirds necrosis in the nephrectomy specimen, compared with patients with other WT subtypes that show less than two-thirds necrosis in response to chemotherapy [20]. In this small ‘proof-of-principle’ case series, we noted that post-operative M-cfDNA levels remained high in the immediate post-operative period, sampled at day 4 up to 24 days post-surgery. This short-term persistence may be due to the fact that serum cfDNA can retain interaction with nucleosome proteins which protects the DNA from degradation, rendering it relatively stable [30]. This may also explain our ability to sequence such a long fragment (319 bp). A full post-surgery time course is required to assess this rate of degradation. Interestingly, one patient showed post-operative M-cfDNA levels 1.3-fold greater than the group mean; a 22% increase post-surgery. We hypothesize that this post-surgery increase in M-cfDNA may be due to residual tumor within the patient and, indeed, three months later, bone metastasis was detected.

## Conclusions

We have defined the first epigenetic biomarker for the analysis of circulating cfDNA in WT patients. We show that this may be useful to improve the accuracy of determining tumor response during pre-operative chemotherapy and predicting the histological risk group. This could allow appropriate modification of treatment prior to planned nephrectomy, particularly important for surgical planning in bilateral WT, where maximizing tumor response to allow partial nephrectomy is the goal. Due to the low relapse rate in WT, a much larger, prospectively collected sample series of patients is required to demonstrate clinical utility as a prognostic biomarker for relapse-free survival. Based on these ‘proof-of-principle’ findings, a European multi-center clinical trial with appropriate sampling is planned to rigorously test whether analysis of this epigenetic biomarker would improve the accuracy of prediction of relapse in all cases.

## Materials and methods

### Sample selection

Use of patient samples in this study was conducted with appropriate parental written consent and ethical approval granted by the NHS London Bridge Research Ethics Committee (reference 12/LO/0101) with experiments performed in compliance with the Helsinki Declaration. Patients were enrolled in the UK either into the SIOP Wilms Tumour 2001 Clinical Trial and Study or the Improving Patient Outcomes for Renal Tumours of Childhood (IMPORT) study, with appropriate parental written consent. Blood serum samples from age-matched controls without cancer were taken from Great Ormond Street Hospital Department of Chemical Pathology with parental written consent. Marmal-aid v1.2.1 [31] was used to extract publically available methylation data annotated as disease = ‘Healthy’ and tissue = blood (n = 411). EK was obtained from fetal post-mortem examinations carried out at the Fetal Pathology Unit, University Hospital of Wales with written parental consent.

### DNA extraction

As NR can only be identified by pathological review of hematoxylin and eosin (H&E)-stained formalin-fixed paraffin embedded (FFPE) sections, 3 μm H&E sections from post-nephrectomy FFPE blocks were studied by two independent pediatric pathologists who marked out regions of NK, NR and WT. A total of 22 matched trios (NK, NR and WT) and 12 matched pairs of NK and WT (90 samples) were microdissected by cutting multiple 5 μm sections and removing the desired region with a scalpel. EB was also microdissected from 5 μm FFPE sections following a master H&E section as a guide. Tissue was taken from the whole section without microdissection to extract DNA from whole EK. Fresh frozen (FF) tissue (n = 86 samples) was taken from 83 patient nephrectomies that were classified according to centralized SIOP pathology review, including stromal (n = 15), epithelial (n = 10), blastemal (n = 11), mixed (n = 23), diffuse anaplastic (n = 14), focal anaplasia (n = 2) or regressive (n = 11) type. DNA was extracted from FFPE and FF tissue using the DNeasy Blood and Tissue Kit (QIAGEN, Hilden, Germany) but with the manufacturer’s instructions modified for FFPE DNA: samples were heated to 90°C for 1 hour post-incubation at 56°C and incubated at 70°C for 10 minutes with buffer AL. For cfDNA analysis, DNA was extracted from pre-chemotherapy (n = 5), pre-operative (n = 8) or post-operative (n = 8) patient serum and age-matched cancer-free control serum (n = 7) using the QIAamp Circulating Nucleic Acid kit (QIAGEN).

### Genome-wide methylation analysis

DNA extracted from FFPE specimens (n = 90) was first treated using the REPLIg FFPE kit (QIAGEN) [32]. Both FF (n = 86) and treated FFPE DNA was then bisulfite-converted using the EZ DNA Methylation Kit (Zymo Research Corp, Orange, CA, USA) and interrogated using the Illumina 450 k platform. Two FFPE NR samples failed stringent quality control metrics and were excluded. For all analyses, open source software packages implemented in R [33] or Bioconductor [34] were used as indicated. Raw data were filtered to exclude samples with detection *P*-value <0.01 and normalized using subset within quantile normalisation (SWAN) using the Bioconductor R package ChAMP version 2.14 [18,35]. For initial MVP detection the normalized data matrix for 22 pairs (NK and WT) was included. Bayesian framework linear modeling using the Bioconductor R package Limma [36] version 3.20.4 [37] was performed to find sites of differential methylation that varied between NK and WT pairs and that were common across patients, which avoids false positives from patient-specific SNPs or age effects. To this model, the TREAT function was applied to adjust *P*-values based on the Δβ-value (>0.1) [38], which were further adjusted to correct for multiple testing [39]. The DMR-lasso algorithm in the ChAMP package was then used to find DMRs [18] with settings adjusted to include only CpGs that reach genome-wide significance (*P* < 5 × 10^-8^). To the same model, we applied the RefFreeEWAS algorithm [19], which uses single value decomposition to estimate the number of cell types contributing to overall histology. The algorithm then deconvoluted the β-values based on the estimated number of cell types (d = 3) and a design matrix specifying patient pairs and sample histology, and generated bootstrap-derived CpG-specific *P*-values and covariates that correspond to a ‘true’ methylation signal with no cell mixture effects.

After confirming our three DMRs were not due to cell composition effects, the DMR values were compared with levels in a replication dataset of 12 pairs, the independent set of 86 FF WT and the matched NRs for 20 cases. Sample classification (by support vector machine) was performed using the 22 pairs as a training set and 12 pairs and 86 WT as separate test sets using R CRAN package e1071 [40].

### Assessment of DMR methylation by bisulfite-sequencing

Of the three DMRs, we chose to validate methylation levels for DMR-1 and -2 by sequencing both bisulfite-converted and normal DNA from nine NK and WT pairs. We performed the same experiment to assess methylation levels in EK and EB. Bisulfite reads for DMR-1 showed poor coverage with only 6/18 validation samples giving sufficient reads (Table S5 in Additional file 2). We therefore chose to focus on DMR-2 for detection in cfDNA from 28 serum samples. For cfDNA analysis, 5/21 patient (3 pre-op, 2 post-op) and 3/7 control samples failed to generate sufficient sequence reads for analysis.

Primers were designed (Table S7 in Additional file 2) using Primer 3 [41] and MethPrimer [42] and optimized using commercial DNA. DNA for bisulfite-sequencing was converted using the EZ DNA methylation kit (Zymo Research). Library preparation PCRs were performed using NEBNext (New England Biolabs, Beverly, MA, USA) and KAPA HiFi Uracil + (KAPA Biosystems Inc, Wilmington, MA, USA). Products were cleaned using magnetic beads (Beckman Coulter Inc, Brea, CA, USA) and quantified using Picogreen reagents. Sample-specific tags were added prior to sequencing using the Illumina Mi-Seq. Raw bisulfite-converted paired-end reads were mapped to human genome build hg19 with Bismark v0.9.0 [43] using Bowtie 2 [44] as the aligner. Methylated and unmethylated base counts were generated with the bismark_methylation_extractor utility and exported as BedGraph files for further analysis and display in IGV [45]. Aligned BAM files were sorted and indexed with SAMtools [46] for assessment of the regions of interest in IGV. The number of C reads divided by total reads per CpG site was then calculated to discern the percentage level of methylation per sample.

To generate allele counts for the full sequence of DMR-2, we used the ANGSD package [47]. ANOVA was performed in R using Bioconductor package Limma to make all possible contrasts between groups. CpGs were selected for further analysis if the Limma Toptable moderated F score >1, indicating that any of the contrasts between groups were non-zero and if group variance for that CpG was >1.

### Data access

The 450 k methylation data described in this study are available from the Gene Expression Omnibus [48] with accession ID GSE59157.

## Additional files

Additional file 1: Table S1. Clinical information for the discovery cohort (n = 22), validation set 1 (n = 12) and validation set 2 (n = 86). Additional file 2: Figure S1. quantification of cell proportions in each micro-dissected Wilms tumor (WT) section used for DNA extraction in the discovery cohort. **Figure S2:** WT precursor lesions show intermediate methylation at significant differentially methylated regions (DMRs). **Figure S3:** comparison of methylation values assessed by 450 k array and bisulfite sequencing. **Table S4:** fresh frozen WT (n = 86) classified by overall tumor histology with average methylation β-values across all significant DMR CpGs. **Table S5:** validation of 450 k methylation signal by bisulfite-sequencing. **Table S6:** clinical information on patients from which cfDNA was isolated. **Table S7:** list of primers used. Additional file 3: Table S2. A list of all cell-corrected methylation variable positions (n = 937). Additional file 4: Table S3. A list of the CpG islands (n = 483) that contained significantly hypermethylated or hypomethylated cell-corrected MVPs, with the respective number of CpGs per island.

## Abbreviations

bp: base pair
cfDNA: circulating cell-free DNA
DMR: differentially methylated region
EB: embryonic blastema
EK: embryonic kidney
FF: fresh frozen
FFPE: formalin fixed paraffin embedded
H&E: hematoxylin and eosin
IMPORT: Improving Population Outcomes for Children with Renal Tumors
M-cfDNA: methylated cell-free DNA
MHC: major histocompatibility complex
MVP: methylation variable position
NK: normal kidney
NR: nephrogenic rest
PCR: polymerase chain reaction
SIOP: International Society of Paediatric Oncology
WT: Wilms tumor
