# Supplementary material for: Methylome analysis identifies a Wilms tumor epigenetic biomarker detectable in blood
Source: Genome Biol. 2014 Aug 19;15(8):434. doi: 10.1186/s13059-014-0434-y (PMC4310621; doi:10.1186/s13059-014-0434-y)
Supplement: Additional file 2: Figure S1. — quantification of cell proportions in each micro-dissected Wilms tumor (WT) section used for DNA extraction in the discovery cohort. Figure S2: WT precursor lesions show intermediate methylation at significant differentially methylated regions (DMRs). Figure S3: comparison of methylation values assessed by 450 k array and bisulfite sequencing. Table S4: fresh frozen WT (n = 86) classified by overall tumor histology with average methylation β-values across all significant DMR CpGs. Table S5: validation of 450 k methylation signal by bisulfite-sequencing. Table S6: clinical information on patients from which cfDNA was isolated. Table S7: list of primers used. [file 13059_2014_434_MOESM2_ESM.docx]

**Additional file 2: Figure S1: Quantification of cell proportions in each micro-dissected Wilms tumour section used for DNA extraction in the discovery cohort.**

(A) Each haematoxylin and eosin stained section was assessed by a paediatric pathologist who confirmed the proportion of blastema (dark grey), stroma (dark yellow), epithelia (light grey), necrosis (black) or chemotherapy induced changes (CIC; light yellow) present in each sample that was micro-dissected. Samples are labelled by the respective average β-value across all DMR CpGs. (B) Correlative analysis between the β_mean_ values for each Wilms tumour sample and the respective proportion of any of the three main cell types did not show any significant correlation with R^2^ values of 0.11, 0.05 and 0.03 for blastema, epithelia and stroma respectively.

**A**


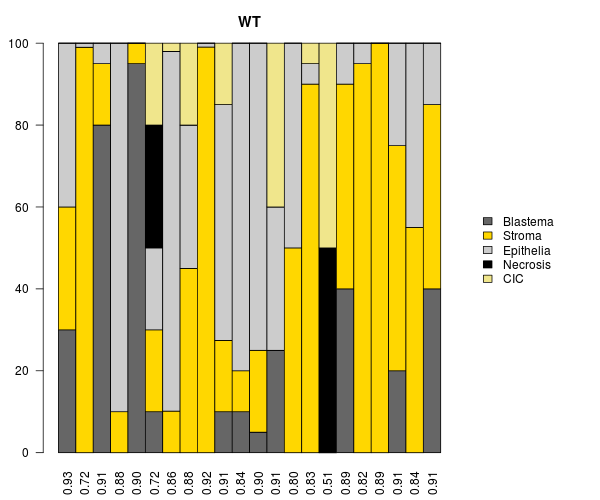


**B**

**hg**

**Additional file 2: Figure S2: WT precursor lesions show intermediate methylation at significant DMRs**

Of the 22 cases where matched normal kidney (NK) and Wilms tumour (WT) was analysed, 20 had nephrogenic rests (NRs) that were also sampled. The average methylation levels for WT (green), NR (orange) and NK (black) across these 20 cases is shown at each DMR with dotted lines representing the standard deviation. In every case, WT is hypermethylated with respect to NK, and the NR appears at an intermediate state of methylation.


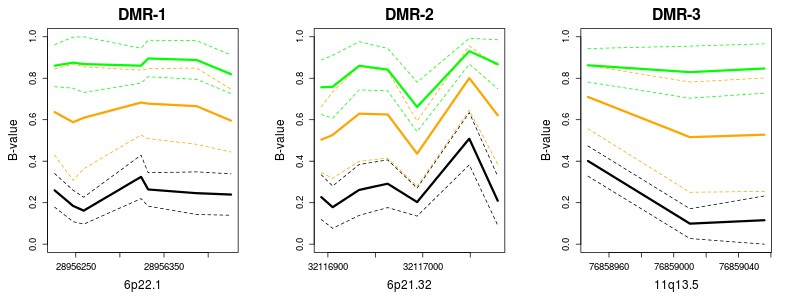


**Additional file 2: Figure S3: Comparison of methylation values assessed by 450k array and bisulfite sequencing**

The β-values discerned by the 450k array (y-axis) compared to level of methylation detected using bisulfite-sequencing (x-axis) show good correlation (Pearson correlation coefficient = 0.884).

**
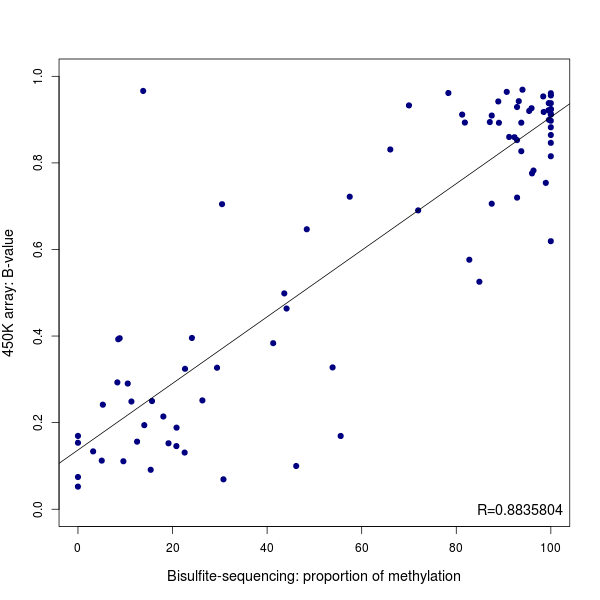
**

**Additional file 2: Table S4: Fresh frozen WT (n = 86) classified by overall tumour histology with average methylation β-values across all significant DMR CpGs**

| **Subtype** | **β_mean_** | **Risk** |
| --- | --- | --- |
| Blastemal.1 | 0.87 | High risk |
| Blastemal.2 | 0.81 | High risk |
| Blastemal.3 | 0.89 | High risk |
| Blastemal.4 | 0.92 | High risk |
| Blastemal.5 | 0.84 | High risk |
| Blastemal.6 | 0.92 | High risk |
| Blastemal.7 | 0.90 | High risk |
| Blastemal.8 | 0.92 | High risk |
| Blastemal.9 | 0.83 | High risk |
| Blastemal.10 | 0.86 | High risk |
| Blastemal.11 | 0.90 | High risk |
| Diffuse_anaplasia.1 | 0.89 | High risk |
| Diffuse_anaplasia.2 | 0.76 | High risk |
| Diffuse_anaplasia.3 | 0.86 | High risk |
| Diffuse_anaplasia.4 | 0.78 | High risk |
| Diffuse_anaplasia.5 | 0.75 | High risk |
| Diffuse_anaplasia.6 | 0.81 | High risk |
| Diffuse_anaplasia.7 | 0.93 | High risk |
| Diffuse_anaplasia.8 | 0.93 | High risk |
| Diffuse_anaplasia.9 | 0.94 | High risk |
| Diffuse_anaplasia.10 | 0.94 | High risk |
| Diffuse_anaplasia.11 | 0.94 | High risk |
| Diffuse_anaplasia.12 | 0.75 | High risk |
| Diffuse_anaplasia.13 | 0.91 | High risk |
| Diffuse_anaplasia.14 | 0.94 | High risk |
| Epithelial.1 | 0.81 | Intermediate risk |
| Epithelial.2 | 0.52 | Intermediate risk |
| Epithelial.3 | 0.75 | Intermediate risk |
| Epithelial.4 | 0.89 | Intermediate risk |
| Epithelial.5 | 0.93 | Intermediate risk |
| Epithelial.6 | 0.82 | Intermediate risk |
| Epithelial.7 | 0.86 | Intermediate risk |
| Epithelial.8 | 0.76 | Intermediate risk |
| Epithelial.9 | 0.91 | Intermediate risk |
| Epithelial.10 | 0.88 | Intermediate risk |
| Focal_anaplasia.1 | 0.73 | Intermediate risk |
| Focal_anaplasia.2 | 0.83 | Intermediate risk |
| Mixed.1 | 0.75 | Intermediate risk |
| Mixed.2 | 0.87 | Intermediate risk |
| Mixed.3 | 0.20 | Intermediate risk |
| Mixed.4 | 0.92 | Intermediate risk |
| Mixed.5 | 0.80 | Intermediate risk |
| Mixed.6 | 0.84 | Intermediate risk |
| Mixed.7 | 0.70 | Intermediate risk |
| Mixed.8 | 0.90 | Intermediate risk |
| Mixed.9 | 0.81 | Intermediate risk |
| Mixed.10 | 0.92 | Intermediate risk |
| Mixed.11 | 0.85 | Intermediate risk |
| Mixed.12 | 0.63 | Intermediate risk |
| Mixed.13 | 0.79 | Intermediate risk |
| Mixed.14 | 0.70 | Intermediate risk |
| Mixed.15 | 0.74 | Intermediate risk |
| Mixed.16 | 0.84 | Intermediate risk |
| Mixed.17 | 0.74 | Intermediate risk |
| Mixed.18 | 0.74 | Intermediate risk |
| Mixed.19 | 0.89 | Intermediate risk |
| Mixed.20 | 0.78 | Intermediate risk |
| Mixed.21 | 0.87 | Intermediate risk |
| Mixed.22 | 0.88 | Intermediate risk |
| Mixed.23 | 0.90 | Intermediate risk |
| Regressive.1 | 0.46 | Intermediate risk |
| Regressive.2 | 0.95 | Intermediate risk |
| Regressive.3 | 0.39 | Intermediate risk |
| Regressive.4 | 0.71 | Intermediate risk |
| Regressive.5 | 0.56 | Intermediate risk |
| Regressive.6 | 0.95 | Intermediate risk |
| Regressive.7 | 0.62 | Intermediate risk |
| Regressive.8 | 0.59 | Intermediate risk |
| Regressive.9 | 0.72 | Intermediate risk |
| Regressive.10 | 0.89 | Intermediate risk |
| Regressive.11 | 0.88 | Intermediate risk |
| Stromal.1 | 0.81 | Intermediate risk |
| Stromal.2 | 0.82 | Intermediate risk |
| Stromal.3 | 0.65 | Intermediate risk |
| Stromal.4 | 0.74 | Intermediate risk |
| Stromal.5 | 0.78 | Intermediate risk |
| Stromal.6 | 0.83 | Intermediate risk |
| Stromal.7 | 0.91 | Intermediate risk |
| Stromal.8 | 0.89 | Intermediate risk |
| Stromal.9 | 0.88 | Intermediate risk |
| Stromal.10 | 0.77 | Intermediate risk |
| Stromal.11 | 0.85 | Intermediate risk |
| Stromal.12 | 0.72 | Intermediate risk |
| Stromal.13 | 0.91 | Intermediate risk |
| Stromal.14 | 0.79 | Intermediate risk |
| Stromal.15 | 0.80 | Intermediate risk |

**Additional file 2: Table S5: Validation of 450k methylation signal by bisulfite-sequencing**

| **DMR** | **Samples with >10 reads** | **Correlation coefficient compared to 450k array** | **Mean difference between 450k value and bisulfite sequencing** | **Range of difference between 450k value and sequencing value** |
| --- | --- | --- | --- | --- |
| 1 | 6/18 | 0.92 | 0.065 | 0.01-0.22 |
| 2 | 16/18 | 0.98 | 0.073 | 0.02-0.16 |

**Additional file 2: Table S6: Clinical information on patients from which cfDNA was isolated**

|  |  |  |  |  | **After pathological review of excised tumour** | | |
| --- | --- | --- | --- | --- | --- | --- | --- |
| **Patient** | **%M_mean_** | **Time point** | **Laterality** | **Days post-surgery serum taken** | **% of chemotherapy induced changes** | **Stage** | **Tumour subtype** |
| Control 1 | 13.3 |  |  |  |  |  |  |
| Control 2 | 8.5 |  |  |  |  |  |  |
| Control 3 | 12.3 |  |  |  |  |  |  |
| Control 4 | 18.1 |  |  |  |  |  |  |
| 1 | 14.4 | Diagnosis | unilateral |  | 5-10% | 1 | Epithelial |
| 1 | 16.4 | End of pre-op chemo |  |  |  |  |  |
| 2 | 13.9 | Diagnosis | unilateral |  | 70% | 1 | Diffuse anaplastic; later bone metastasis |
| 2 | 20.3 | Mid pre-op chemo |  |  |  |  |  |
| 2 | 24.7 | Start of post-op chemo |  | 20 |  |  |  |
| 3 | 18.2 | Diagnosis | unilateral |  | 60% | 3 | Diffuse anaplastic |
| 4 | 11.0 | Diagnosis | unilateral |  | 20% | 1 | Mixed |
| 5 | 15.0 | Diagnosis | bilateral |  | right: 40%  left upper: 0%  left lower: 80% | 5 | right: mixed  left upper: mixed  left lower: regressive |
| 5 | 18.5 | Start of post-op chemo |  | 13 |  |  |  |
| 6 | 15.4 | End of pre-op chemo | unilateral |  | 2-3% | 3 | Diffuse anaplastic and blastemal |
| 6 | 16.3 | Start of post-op chemo |  | 8 |  |  |  |
| 7 | 22.2 | End of pre-op chemo | unilateral |  | 95% | 3 | Regressive |
| 8 | 25.2 | End of pre-op chemo | unilateral |  | 90% | 3 | Regressive |
| 8 | 19.9 | Start of post-op chemo |  | 4 |  |  |  |
| 9 | 19.6 | Start of post-op chemo | unilateral | 24 | 20% | 1 | Mixed |
| 10 | 15.9 | Start of post-op chemo | unilateral | 17 | 5-10% | 1 | Epithelial |

**Additional file 2: Table S7: List of primers used**

| **Region** | **Forward primer** | **Reverse primer** | **Tm** |
| --- | --- | --- | --- |
| DMR1 | TGTCGCCTGCACTAAGAGAA | CGCAGTCCTTGCTGTTTATG | **57** |
|  | CGGGTCACCTTCTGAATTTT | TTGCGTCACCGATGTAAGAG | **57** |
| DMR2 | CGATGTATCCAAGTCTGACG | ACTTCTCCTTCATCTCCCTG | **58** |
| DMR1 bisulfite-converted | TTTTTATTTTTATAAATAGTAAGGATTG | AAAAACTTCAAACAACTATTCAACC | **51** |
|  | GTTAAAATTTTGGGTTAAGGAGAT | AAAATAAAAATTCAAAAAATAACCC | **51** |
| DMR2 bisulfite-converted | GGTTTTTGAGGAATTGGATTT | TCCCAAACTCTTTCTACAATCATAC | **51** |
|  | ATTGTAGAAAGAGTTTGGGAGAT | TTCTAAAAACCATCTTAAAAAAAA | **51** |
